# The eTM–miR3699–MAN7 mediated cell wall degradation in regulating embryogenic cell formation during the early stage of somatic embryogenesis in apple

**DOI:** 10.1093/hr/uhaf315

**Published:** 2025-11-14

**Authors:** Yue Yang, Yu Wang, Mingkun Chen, Xilin Zhou, Jun Wei, Jiayao Tang, Houhua Li

**Affiliations:** College of Landscadpe Architecture and Art, Northwest A&F University, Yangling, Shaanxi 712100, China; College of Landscadpe Architecture and Art, Northwest A&F University, Yangling, Shaanxi 712100, China; College of Landscadpe Architecture and Art, Northwest A&F University, Yangling, Shaanxi 712100, China; College of Horticulture and Forestry, Tarim University, Alar, Xinjiang 843300, China; College of Landscadpe Architecture and Art, Northwest A&F University, Yangling, Shaanxi 712100, China; College of Landscadpe Architecture and Art, Northwest A&F University, Yangling, Shaanxi 712100, China; College of Landscadpe Architecture and Art, Northwest A&F University, Yangling, Shaanxi 712100, China

## Abstract

Somatic embryogenesis (SE) in plants requires the prior formation of embryogenic cells in plants. The remodeling of the cell wall in mature somatic cells is a prerequisite for embryogenic cell formation. However, the mechanism of this process remains unelucidated. In this study, eTM3699, miR3699, and *MANNAN7* (*MAN7*) were identified as key regulators of embryogenic cell formation through whole-transcriptome sequencing. The dual-luciferase reporter assays and GUS histochemical staining assays, were used to identified the regulatory network of eTM3699–miR3699–*MdMAN7*. The overexpression and CRISPR/Cas9-mediated transgenic assays were used for functional analysis of miR3699 and *MdMAN7*. *MdMAN7* overexpression can enhance the activity of β-mannanase, induce hemicellulose degradation, reshape the cell wall of highly differentiated somatic cells, and relieve the restriction on cell differentiation and division, ultimately positively regulating the embryogenic cell formation. Specifically, the overexpression of *MdMAN7* can significantly improve the efficiency and shorten the induction cycle of SE. miR3699 acted by negatively regulating *MdMAN7.* In addition, eTM3699 were identified as endogenous target mimics of miR3699 that bind to miR3699 to prevent cleavage of *MdMAN7* and thereby positively regulate embryogenic cell formation. In conclusion, our results elucidate the mechanism of eTM–miR3699–*MAN7* module regulating embryogenic cell formation during the early stage of SE in apple.

## Introduction

Somatic embryogenesis (SE) is characterized by a low mutation rate, short induction period, and high reproductive coefficient, making it an excellent receptors for genetic transformation and suitable model for cell reprogramming mechanistic studies in plants [1]. SE fundamentally involves reprogramming somatic cells to alter their developmental fate, which fully reflects the pluripotency of plant cells. The dedifferentiation of mature somatic cells and then transformed into embryonic cell is a prerequisite for SE. The cell wall’s mechanical and chemical cues are key regulators of this process [2]. However, the mechanism of cell wall modification in embryonic cell formation remains unclear.

The cell wall are not only inert structures surrounding plant cells, but also involved in regulating development in plants, such as signal transmission, stress response, and cell elongation and division [3–5]. The differentiation of plant cells were controlled by the position effects. If the position of the cell was changed in an appropriate way, the differentiation fate of this cell will also change accordingly. Furthermore, the cell wall is crucial in determining the fate of cell differentiation through the formation of positional effects [6]. For example, the study on the formation and stability of polar axis in *Fucus vesiculosus* showed that although protoplasts (without cell wall) can respond light signals to form polar axis, they are not stable. The light-induced polar axis can be stably maintained only when the cell wall exists. Moreover, the protoplasts with regenerated cell wall can divide and differentiate like normal zygotes, which indicated that the cell wall not only serves as the physical foundation for stabilizing the polar axis but is also essential for maintaining the differentiated cell state [7]. The subsequent study on root hair regeneration has confirmed the above conclusions in *Arabidopsis thaliana* [8]*.*

The dedifferentiation of highly differentiated somatic cells into embryogenic cells is a complex process in plants. The results of early study on SE in carrot indicated that localized degradation of the cell wall might be a prerequisite for cell dedifferentiation [9]. Subsequently, it has been discovered that exogenous application of chitinase can restore the embryogenic ability in carrot mutants, indicating that dynamic modification of cell wall components is crucial for activating pluripotency [10]. During the early stage of SE in carrot, partial degradation of the cell wall releases oligosaccharide signaling molecules, which subsequently activate the cell reprogramming and induce somatic cells pluripotency expression [11]. Moreover, the cell wall-related proteins (expansins) cause the degradation of hemicellulose, thereby reducing cell wall rigidity, which directly promotes cell dedifferentiation in *Medicago truncatula* [12]. Although several studies have been reported that the cell dedifferentiation regulated by cell wall [13, 14], the mechanism is still limited.

The primary components of the dicotyledonous plant cell wall are cellulose, hemicellulose, pectin, and protein. Mannan, a polysaccharide, is a key component of hemicellulose [15]. *MANNAN* (*MAN*) gene family modulates β-1,4-mannanase activity to promote cell wall degradation, thereby participating in processes such as cell expansion [16] and fruit ripening [17] in plants. However, the function and mechanism of *MAN* in SE, particularly in the formation of embryogenic cells remains unclear, and required further investigation.

In recent years, many small noncoding RNAs (miRNAs) have been identified as key regulators for SE in plants through targeted inhibition of mRNA expression, such as miR156-*SPLs* [18], miR171-*SCLs* [19], miR408-*DlNUDT23* [20, 21], and miR858-*MYBs* [22]. In addition, long non-coding RNAs (lncRNAs) can act as miRNA endogenous target mimics (eTMs), sequestering miRNAs and preventing their interaction with target mRNAs. This competitive binding attenuates miRNA-dependent gene silencing [23]. The competing endogenous RNA (ceRNA) hypothesis proposes that the cross-talk between lncRNAs, miRNAs, and mRNAs is a core regulatory mechanism of gene expression [24]. For examples, in *Lilium pumilum* DC. Fisch, the eTM171 promotes SE by binding to miR171, thereby counteracting the inhibitory effect of miR171 on target gene *SCL6* [19]. The eTM354 can bind to miR160b to response the salt stress in cotton [25]. To date, the ceRNA regulation of SE in plants is still limited.

This study identified miR3699 as a potential key regulator in the formation of embryogenic cells under the induction of auxin through whole-transcriptome sequencing. Moreover, the lncRNA (MSTRG.1226.2) act as endogenous target mimics (eTM3699) of miR3699 was screened, which compete with target gene *MdMAN7* to regulate the cell wall mediated embryogenic cell formation in apple. The mechanism of eTM-miR3699-*MdMAN7* is a novel regulatory module in SE in apple. The results will provide a theoretical basis for the mechanism of ceRNA mediated cell wall modification in somatic cell reprogramming in plants.

## Results

### The formation of embryogenic cell and SE is induced by exogenous auxin in apple

**Figure 1:**
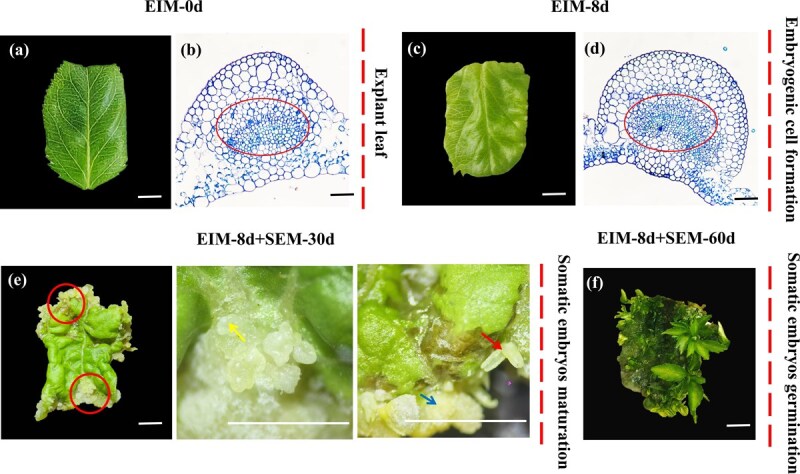
Somatic embryo induction and histological observation in apple. (a) Explant leaf, bar = 5 mm. (b) Histological observation of uninduced apple leaf, circle, morphological changes in cells near veins, scale bar = 20 μm. (c) The explant leaf at 8 days of EIM cultivation, bar = 5 mm. (d) Many embryogenic cells that were dividing vigorously were observed at 8 days of EIM cultivation, circle, morphological changes in cells near veins, scale bar = 20 μm. (e) The explant leaf cultured in EIM for 8 days and SEM for 30 days, embryonic callus, Torpedo embryo, cotyledonary embryo, bar = 5 mm. (f) The explant leaf cultured in EIM for 8 days and SEM for 60 days, regenerated plantlets, bar = 5 mm.

The entire process of SE in apple is shown in Fig. 1. The embryonic induction medium (EIM, MS semisolid medium with 0.5 mg·l^−1^ 2,4-D and 3.0 mg·l^−1^ NAA, pH 5.8 ± 0.2) was used to induce embryogenic cell formation and the somatic embryogenesis medium (SEM, MS semisolid medium with 1.0 mg·l^−1^ 6-BA, pH 5.8 ± 0.2) was used to induce somatic embryo maturation and germination. The explant leaves were cut into rectangle to induce SE (Fig. 1a). On the Day 8 of auxin induction, the irregular protrusions appeared on the surface of the leaves (Fig. 1c). The histological and cytological observations were performed to identify the cell state of the longitudinal section around the veins. Compared with the induction of auxin on Day 0 (Fig. 1b), a marked proliferation of smaller, densely packed cells occurred in the xylem and phloem on Day 8, and were in a vigorous state of division (Fig. 1d), which indicated the formation of embryonic cells under the induction of auxin on Day 8. The embryonic leaves were subsequently transferred to SEM for 30 days at darkness until the mature cotyledonary embryos formed on the leaves (Fig. 1e). The mature cotyledonary embryos were subsequently transferred to 16/8 h light/dark conditions for more than 30 days to induce the embryos germination (Fig. 1f).

### Identification of differentially expressed lncRNAs and mRNAs

**Figure 2:**
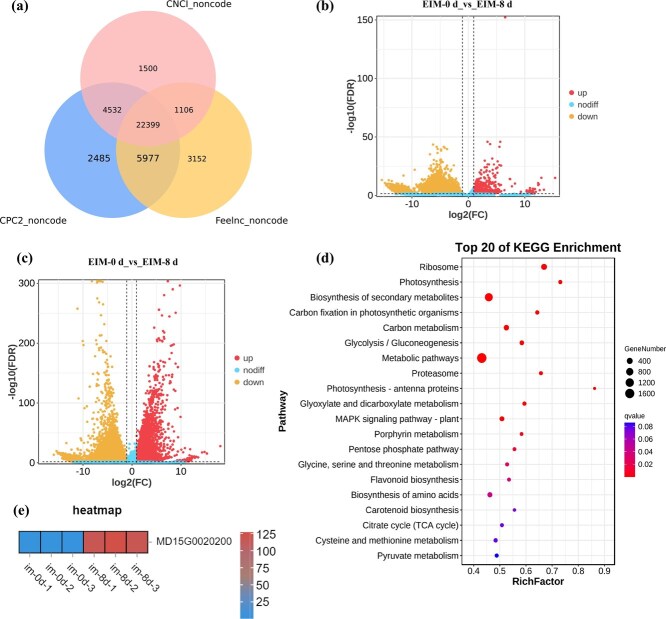
Identification of differentially expressed lncRNAs and mRNAs involved in embryogenic cell formation in *M. domestica* ‘Gala.’ (a) Initial lncRNA identification was conducted with CNC1, CPC2, and Feelnc. (b) DElncRNAs volcano plot. (c) DEmRNAs volcano plot. (d) KEGG enrichment analysis of DEmRNAs. (e) Heatmap of *MdMAN7* (MD15G0020200) under the induction of auxin on Day 0 and Day 8.

Transcript assembly revealed 35 468 lncRNAs in the early stages of SE (0 and 8 days) in apple (Fig. 2a), which were classified into six categories: 1331 sense lncRNAs, 4592 antisense lncRNAs, 1851 intronic lncRNAs, 1003 bidirectional lncRNAs, 11 677 intergenic lncRNAs, and 1945 other types (Fig. S1). In total, 17 133 (Table S2) DElncRNAs were detected, including 16 612 upregulated and 521 downregulated lncRNAs (Fig. 2b).

A total of 23 633 mRNAs (Table S3) were identified as differentially expressed (16 893 upregulated and 6740 downregulated) between the induction of auxin on Day 0 and Day 8 (Fig. 2c). Gene Ontology (GO) enrichment analysis showed significant representation in 51 functional terms across three major categories (Fig. S2), and Kyoto Encyclopedia of Genes and Genomes (KEGG) analysis revealed that the differentially expressed genes (DEGs) are involved in metabolic pathways (Fig. 2d). The significant enrichment of plant hormone signal transduction pathways, particularly auxin and cytokinin, among the DEGs underscores the pivotal role of phytohormone crosstalk in re-initiating a totipotent state during SE. Concurrently, the enrichment of starch and sucrose metabolism likely reflects the substantial energy and carbon skeleton demands for building new embryonic structures, indicating a reprogramming of central metabolism to fuel embryogenesis.

The degradation of the cell wall is a prerequisite for the dedifferentiation of mature somatic cells. A significant enrichment of genes encoding cell wall degradation enzymes, such as *MANNAN* (*MAN*), *XyloGlucan transglycosylase/Hydrolase* (*XTH*), *Cellulase* (*CEL*), and *Pectate Lyase* (*PGL*), were observed in the metabolic pathways. Among these, *MdMAN7* showed the most significant differential expression, exhibiting a 55-fold increase under auxin treatment (Table S3). Furthermore, miRNA-mRNA association analysis using the patmatch_v1.2 software revealed that *MdMAN7* (MD15G0020200) was the most significantly and confidently predicted target of the core regulatory module, miR3699, which itself was strongly differentially expressed. Thus, *MdMAN7* was selected as the central regulator related to cell wall degradation during embryogenic cell formation in apple for subsequent regulatory network and functional analysis.

### Analysis of differentially expressed miRNAs and targeted genes

**Figure 3:**
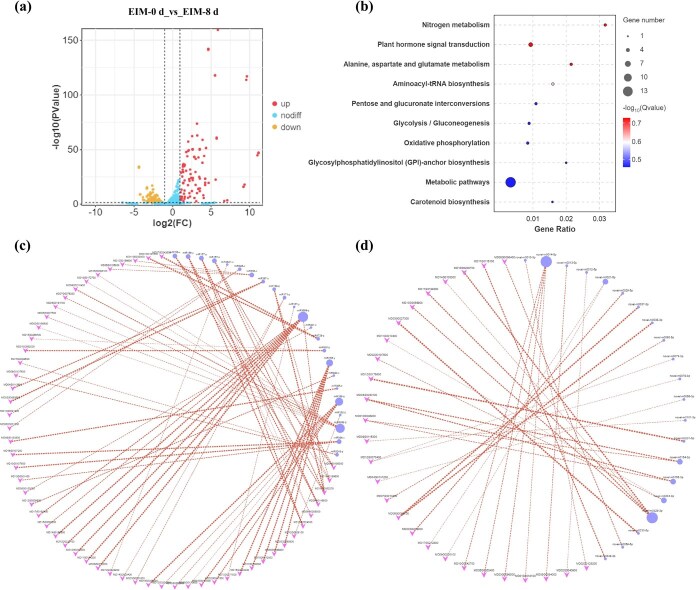
Function analysis of DEmiRNAs and their target genes in *M. domestica* ‘Gala.’ (a) DEmiRNAs volcano plot. (b) KEGG pathway enrichment analysis of miRNA-targeted differentially expressed mRNAs. (c) The network diagram of targeted relationship between known DEmiRNAs and DEmRNAs. (d) The network diagram of targeted relationship between novel DEmiRNAs and DEmRNAs.

A total of 6574 potential miRNAs were identified in *Malus domestica* ‘Gala’ leaves, including 1868 existing miRNAs, 1264 known miRNAs, and 3442 novel miRNAs. The identified miRNAs ranged from 18 to 24 nt, predominantly 24 nt (Fig. S5). There were 295 DEmiRNAs (117 upregulated, 178 downregulated) (Table S4) in the group EIM_0 d and EIM_8 d (Fig. 3a). Target mRNA analysis showed enrichment in 41 GO terms across three categories (Fig. S4), with the top 10 KEGG pathways including plant hormone signaling and metabolic pathways (Fig. 3b), which indicated that the differences in metabolites mediated by auxin signaling are key factors in the formation of embryonic cells in apple. A total of 95 pairs of miRNAs with their target genes were predicted, including 64 pairs of known miRNAs with target genes (Fig. 3c) and 31 pairs of novel miRNAs (Fig. 3d).

### Construction of competitive endogenous RNA network

**Figure 4:**
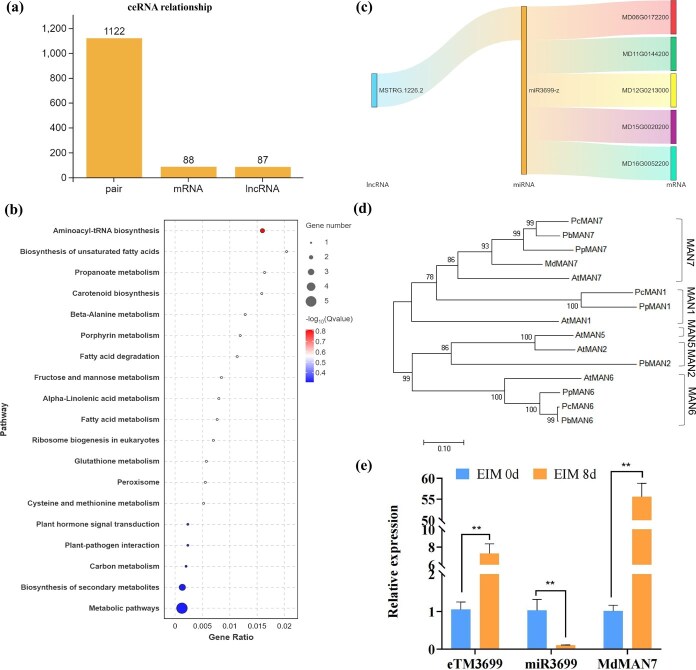
Identification of ceRNAs in the early stage of SE in *M. domestica* ‘Gala.’ (a) The ceRNA relationships under the treatment of auxin in *M. domestica* ‘Gala.’ (b) The KEGG pathway enrichment of ceRNAs. (c) The eTM3699–miR3699–*MdMAN7* module diagram. (d) Phylogenetic tree analysis of *MdMAN7.* (e) RT-qPCR analysis of eTM3699, mature miR3699 and *MdMAN7* in the early stage of SE in *M. domestica* ‘Gala.’ Values represent means ± SEs. Statistical significance was assessed by Student’s *t*-test (^*^P < 0.05, ^**^P < 0.01).

Based on the ceRNA hypothesis, the 1122 pairs of ceRNA relationships were retrieved, comprising 88 DEmRNAs and 87 DElncRNAs interactions (Fig. 4a). GO enrichment revealed that 19 significant terms across three categories for the ceRNAs (Fig. S4). The top 20 enriched KEGG pathways included metabolic pathways (Fig. 4b). We focused on screening potential cell differentiation factors, and the *MdMAN7* (MD15G0020200) was speculated to be a key regulator through DEmRNA analysis, thereby the miRNA3699–*MdMAN7* pair was screened. The lncRNA MSTRG.1226.2 (eTM3699) was predicted to bind to miR3699. Therefore, the ceRNA network eTM3699–miR3699–*MdMAN7* was mapped (Fig. 4c). Phylogenetic tree analysis showed that *MdMAN7* is closely related to *PcMdMAN7*, *PbMdMAN7*, and *PpMdMAN7,* which indicated that the phylogenetic relationship between apple and pear is close (Fig. 4d). Moreover, RT-qPCR analysis was performed to identify the eTM3699–miR3699–*MdMAN7* module. The eTM3699 and *MdMAN7* expression increased significantly by 7- and 55-fold under the treatment of auxin, respectively, whereas mature miR3699 expression levels decreased by 10-fold (Fig. 4e). eTM3699 and *MdMAN7* exhibited consistent expression patterns, while miR3699 showed an opposite relationship, which is consistent with ceRNA regulatory principles. This results indicated that eTM3699–miR3699–*MdMAN7* are involved in SE and may play an important role in the embryogenic transition in apple.

### Cytological observation and the content of cell wall-related metabolites during embryonic cell formation

**Figure 5:**
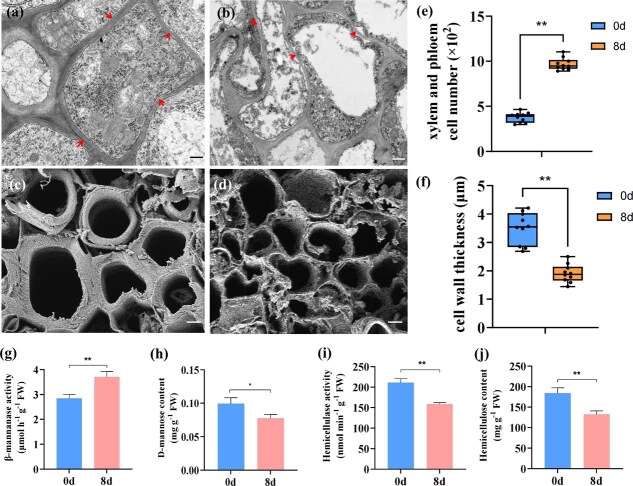
Cytological observation and the detection of physiological-biochemical related to cell wall synthesis during the formation of embryogenic cells in *M. domestica* ‘Gala.’ (a) Observation of mature somatic cells by transmission electron microscopy on EIM_0 d, bar = 5 μm. (b) Observation of embryogenic cells by transmission electron microscopy by electron microscopy on EIM_8 d, bar = 5 μm. (c) Observation of mature somatic cells by scanning electron microscopy on EIM_0 d, bar = 10 μm. (d) Observation of embryogenic cells by scanning electron microscopy on EIM_8 d, bar = 10 μm. (e) The number of xylem and phloem cells of the transversal section around the leaf veins on EIM_0 d and EIM_8 d. (f) The cell wall thickness of mature somatic cells and embryogenic cells. (g) The activity of β-mannanase in leaves on EIM_0 d and EIM_8 d. (h) The content of D-mannose in leaves on EIM_0 d and EIM_8 d. (i) The activity of hemicellulase in leaves on EIM_0 d and EIM_8 d. (j) The content of hemicellulose in leaves on EIM_0 d and EIM_8 d. Values represent means ± SEs. Statistical significance was assessed by Student’s *t*-test (^*^P < 0.05, ^**^P < 0.01).

To visualize cell wall architecture, scanning and transmission electron microscopy (SEM/TEM) analysis was performed. Mature somatic cells are separated by prominent physical barriers formed by thick cell walls, and various organelles such as the nucleus, mitochondria, and Golgi apparatus are clearly visible (Fig. 5a). However, the cell wall significantly thinned after embryonic differentiation, and vacuole occupies the vast majority of the cellular space, compressing other organelles to the periphery (Fig. 5b). The histological and cytological observations of the transversal section around the leaf veins showed that, compared with the explant leaf on Day 0 (Fig. 5c), smaller cells with compact organization showed a marked numerical increase, and the cell walls of these cells become significantly thinned (Fig. 5d). The number of xylem and phloem cells of the transversal section around the leaf veins exhibited a significant 3.4-fold increase after 8 days of auxin induction (Fig. 5e). Moreover, compared with mature somatic cells, embryonic cells exhibited an average of 1.25-fold reduction in cell wall thickness (Fig. 5f). The physiological-biochemical related to cell wall synthesis results showed that during the formation of embryogenic cells, the activity of β-mannanase significantly increased (Fig. 5g), while the content of D-mannose exhibited decline (Fig. 5h). Additionally, both the activity of hemicellulase (Fig. 5i) and the content of hemicellulose (Fig. 5j) correspondingly demonstrated significant decrease, which indicated that the degradation of cell walls plays a crucial role in the formation of embryogenic cells.

### Identification of eTM3699–miR3699–*MdMAN7* network

**Figure 6:**
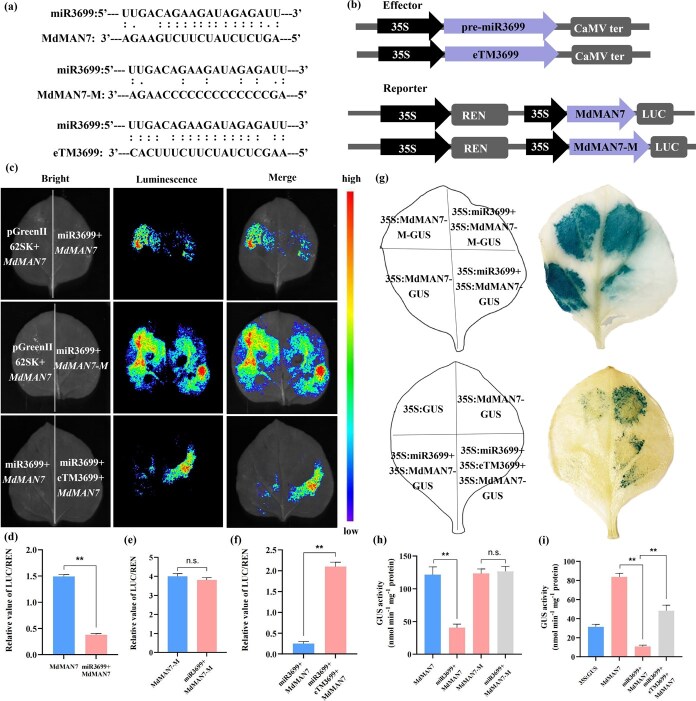
Validating the eTM3699–miR3699–*MdMAN7* interaction. (a) Binding sites of miR3699 in eTM3699 and target gene *MdMAN7. MdMAN7*-M carries the mutant sequence targeted by mature miR3699. (b) Schematic of GUS and dual-luciferase reporter constructs. (c) Dual-luciferase image of miR3699 and MdMAN7-LUC; miR3699 and MdMAN7-M-LUC; eTM3699, miR3699 and MdMAN7-LUC in tobacco leaves. (d) Relative luciferase activity (LUC/REN) of miR3699 and MdMAN7-GUS. (e) Relative luciferase activity (LUC/REN) of miR3699 and MdMAN7-M-GUS. (f) Relative luciferase activity (LUC/REN) of eTM3699, miR3699 and MdMAN7-GUS. (g) GUS histochemical staining of tobacco leaves cotransformed with miR3699 and MdMAN7-GUS; miR3699 and MdMAN7-M-GUS; eTM3699, miR3699, and MdMAN7-GUS. (h and i) Quantification of β-glucuronidase activity in tobacco leaves shown in (g). Values represent means ± SEs. Statistical significance was assessed by Student’s *t*-test (^*^P < 0.05, ^**^P < 0.01). n.s., no significant difference.

The lncRNA (MSTRG.1226.2) was predicted by _PS_RNAT_ARGET_ (https://www.zhaolab.org/psRNATarget/) to function as an endogenous target mimic (eTM3699) of miR3699, which compete with target gene *MdMAN7* to regulate the cell wall mediated embryogenic cell formation. The *MdMAN7* mutant sequence, which is non-complementary to mature miR3699, was synthesized to identify the ceRNA network. The targeting–matching relationships in eTM3699 and miR3699 as well as miR3699 and *MdMAN7* were illustrated in Fig. 6a. GUS and dual-LUC reporter/effector vectors were constructed (Fig. 6b). The fluorescence intensity significantly decreased when miR3699 and MdMAN7-LUC were co-expressed compared with the control (Fig. 6c and d), which indicated that miR3699 can inhibit the transcription of *MdMAN7.* No significant difference in fluorescence intensity was observed when miR3699 and MdMAN7-M-LUC were co-expressed compared with the control (Fig. 6c and e), which indicated that mature miR3699 is targeting the sequence of *MdMAN7.* The fluorescence intensity significantly increased after eTM3699 was added to the combination of miR3699 and MdMAN7-LUC (Fig. 6c and f), indicating that eTM3699 inhibits miR3699-mediated cleavage of *MdMAN7*, as further validated by LUC/REN assays (Fig. 6d–f). Furthermore, GUS staining intensity remained unchanged when tobacco leaves were cotransformed with miR3699 and *MdMAN7*-M-GUS relative to the control, while the staining intensity significantly decreased when miR3699 and MdMAN7-GUS were co-expressed compared with the control. The staining intensity significantly increased after eTM3699 was added to the combination of miR3699 and MdMAN7-GUS (Fig. 6g). Quantification of GUS enzyme activity confirmed the staining intensity patterns observed in the coloration assays (Fig. 6h and i). Collectively, these results establish eTM3699 as the eTM of miR3699, which competitively binds the miRNA to block its cleavage of *MdMAN7* transcripts*.*

### Functional analysis of miR3699 and *MdMAN7* in embryogenic cell formation in *M. domestica* ‘Gala’

miR3699 and *MdMAN7* overexpression lines were obtained to analyze the gene functions (Fig. S6a). The expression levels of mature miR3699 and *MdMAN7* in the OE-miR3699 and OE-*MdMAN7* lines were significantly upregulated through RT-qPCR results. Transgenic lines *#*OE-miR3699-3/4 and *#*OE-*MdMAN7-*2/5 served as the foundation for functional analysis (Fig. S6b and c). The fragment of *HYG* was amplified to revealed the CRISPR/Cas9-mediated transgenic positive plants were obtained (Fig. S7a). As a result, the *#*KO-*MdMAN7-*2/3 with nucleotide replacements, insertions, and deletions caused frame shifts and premature termination of translation (Fig. S7b and c).

**Figure 7:**
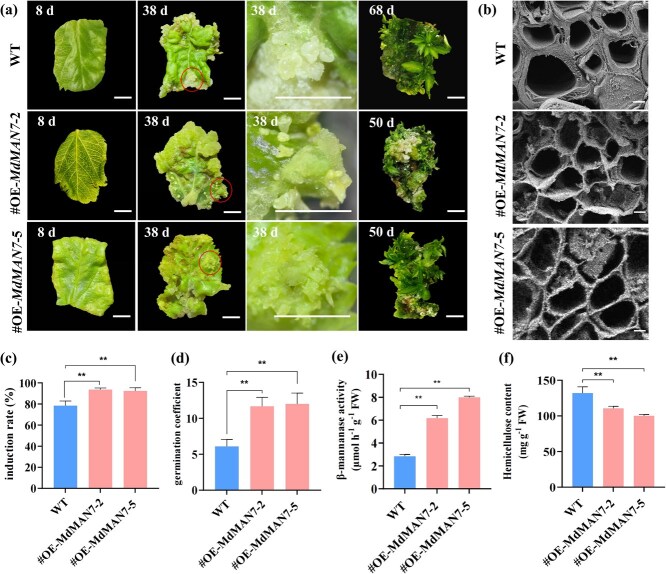
Functional analysis of OE-*MdMAN7* transgenic lines of SE in apple. (a) The SE and germination of the WT and OE-*MdMAN7* apple lines, bar = 5 mm. (b) Observation of cell walls by scanning electron microscopy in the WT and OE-*MdMAN7* lines, bar = 10 μm. (c) Somatic embryo induction rates under SEM for 30 days. (d) Germination coefficients under SEM were determined at ~42 d for OE-*MdMAN7* lines and ~60 d for WT. (e) The activity of β-mannanase in the WT and OE-*MdMAN7* lines. (f) The content of hemicellulose in the WT and OE-*MdMAN7* lines. Values represent means ± SEs. Statistical significance was assessed by Student’s *t*-test (^*^P < 0.05, ^**^P < 0.01).

Overexpression of *MdMAN7* significantly promote SE in *M. domestica* ‘Gala.’ OE-*MdMAN7* lines produced significantly more mature somatic embryos than wild type (WT) after 30 days in SEM. Furthermore, following accelerated germination of somatic embryos (~50 d in OE-*MdMAN7* lines vs. ~68 d in WT), the OE-*MdMAN7* lines exhibited a significantly higher plant regeneration efficiency, yielding an average of 12 plants per unit compared to 6 in the WT. (Fig. 7a). The results of scanning electron microscope showed that compared with the WT, the cell walls of cells that are adjacent to leaf veins become significantly thinned in the OE-*MdMAN7* lines (Fig. 7b). The somatic embryo induction rates of the OE-*MdMAN7* transgenic plants increased from 78% to 95% (Fig. 7c), and the germination coefficient increased from 6 to 12 (Fig. 7d). The physiological-biochemical related to cell wall synthesis results showed that the activity of β-mannanase significantly average increased by 2.5 fold in the OE-*MdMAN7* lines (Fig. 7e), which leads to a significant decrease in hemicellulose content by 0.85 fold the original level (Fig. 7f).

**Figure 8:**
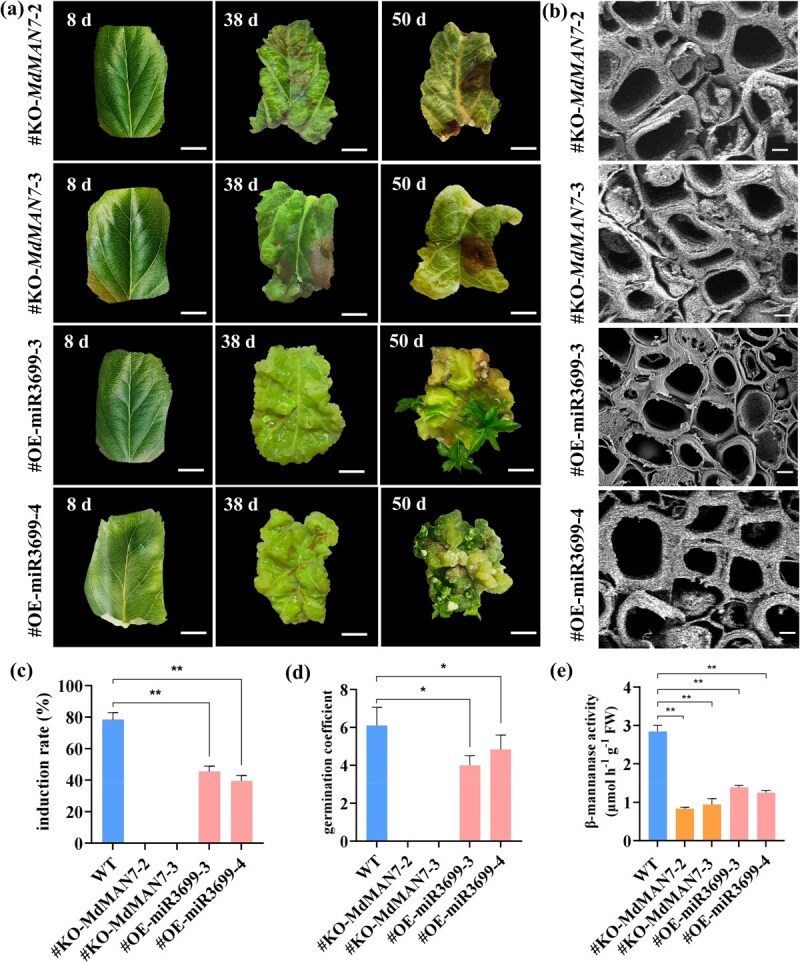
Functional analysis of KO-*MdMAN7* and OE-miR3699 transgenic lines of SE in apple. (a) The SE and germination of the KO-*MdMAN7* and OE-miR3699 apple lines, bar = 5 mm. (b) Observation of cell walls by scanning electron microscopy in the KO-*MdMAN7* and OE-miR3699 lines, bar = 10 μm. (c) Somatic embryo induction rates under SEM for 30 days. (d) Germination coefficients under SEM were determined at ~42 d for OE-*MdMAN7* lines and~60 d for WT. (e) The activity of β-mannanase in the KO-*MdMAN7* and OE-miR3699 lines. Values represent means ± SEs. Statistical significance was assessed by Student’s *t*-test (^*^P < 0.05, ^**^P < 0.01).

Knocking out *MdMAN7* abolished SE and plant regeneration. The leaves turned brown and without any somatic embryos or regenerated plants after induction in SEM (Fig. 8a). Overexpression of miR3699 significantly inhibit SE in *M. domestica* ‘Gala.’ There was only a small number of callus and somatic embryos in OE-miR3699 lines after 30 days of induction in SEM, and a limited number of regenerated plants with stunted and slender growth after 42 days of induction in SEM (Fig. 8a). The results of scanning electron microscope showed no significant difference in the cell walls thickness between WT, KO-*MdMAN7*, and OE-miR3699 lines (Fig. 8b). The somatic embryo induction rates of the KO-*MdMAN7* and OE-miR3699 transgenic plants correspondingly decreased from 78% to 0–42% (Fig. 8c), and the germination coefficient decreased from 6 to 0–5 (Fig. 8d). The activity of β-mannanase significantly decreased by 0.32–0.46 fold in the KO-*MdMAN7* and OE-miR3699 lines (Fig. 7e). These findings collectively demonstrate that *MdMAN7*-mediated cell wall synthesis plays positive effects on embryogenic cell formation and SE in apple. Conversely, miR3699 exerts negative effects on these processes by targeting and suppressing *MdMAN7* expression.

## Discussion

When newly divided plant cells enter the early stages of differentiation, cell wall formation begins. When the primary wall proves insufficient to support the cell structure, the secondary wall begins to form. The thickening of the secondary cell wall results in highly differentiated plant cells possessing structurally reinforced cell walls, which thereby restricts the capacity of highly differentiated cells to dedifferentiate [26]. Therefore, alleviating the constraints imposed by the rigid cell wall structure is a necessary condition for the dedifferentiation of plant somatic cells. The composition and abundance of cell wall components exhibit species- and tissue-specific diversity [27]. The balance of pectin methylesterases– and pectin methylesterase inhibitors–mediated cell wall homeostasis is essential for early stage of SE in *A. thaliana* [28], *Quercus suber* L*.* [29], and *Quercus alba* [30]. Arabinogalactan proteins (AGPs) were also detected in cell walls of proembryogenic masses and somatic embryos [29]. A large amount of AGPs was detected in the cell walls covering the somatic embryos of the highly embryogenic cell line AC78, while the content of AGPs was extremely low in the low-embryogenic cell line AC77 in *Abies alba* × *Abies cephalonica* [31]. AGPs are also specifically deposited in the embryogenic callus in *A. thaliana* [32]. These findings indicate that the formation of SE is closely related to the chemical composition of the cell wall in plants. Furthermore, AGPs also as a signal molecule in the process of androgenesis [33]. β-1,3-glucanases, chitinases, and AGPs are widely distributed in the vesicles and inner cell walls of microspores, as well as in the cell walls of anther cell layers. These components are crucial for inducing the early stages of androgenesis [34]. These findings indicate that cell wall components are also highly important for embryogenesis. Mannose is one of the primary constituents involved in forming the backbone of hemicellulose [35]. Mannans, as hemicellulosic polysaccharides found in plant cell walls, function as storage [36], structural substances [37], and signaling molecules in cell differentiation [38]. This study demonstrate that during the stage of somatic cells transform into embryogenic cells (EIM 0–8 days), the activity of β-mannanase significantly increased, leading to a decrease in D-mannose and hemicellulose content, accompanied by cell wall thinning. Furthermore, compared with the WT, the overexpression lines that enhanced somatic embryogenic capacity exhibit elevated β-mannanase activity, reduced hemicellulose content, and thinner cell walls. Collectively, these findings indicate that cell wall degradation is essential for the initiation of the embryonic program in cells, and β-mannanase plays a crucial role in this process.

The *LeMAN1* was the first mannanase gene, which was cloned from tomato [39]. Subsequently, functional analysis of mannanase genes has been carried out in plants. *MAN* gene family participate in developmental processes by hydrolyzing the mannan backbone within the cell wall. For examples, *LeMAN1* localizes to the tomato seed endospermis, providing energy and carbon sources for seedling growth [40]. *LeMAN2* mediates micropylar endosperm softening to facilitate germination [41]. *LeMAN4* encodes a cell wall hydrolase and functions in fruit ripening and softening [42]. In Arabidopsis, *MAN* genes participate in various developmental processes. For examples, the hydrolysis of mannan-rich endosperm cell walls during *A. thaliana* seed germination is mediated by key enzymes including *AtMAN5*, *AtMAN6*, and *AtMAN7* [43]. *MAN* genes enhance salt tolerance by promoting lateral root development, through its roles in initiating lateral root primordia via the IAA-ARF pathway and modulating ROS homeostasis in Arabidopsis [20, 21]. The functions of *MAN* genes have been extensively studied in tomato and Arabidopsis, while the studies on *MAN* genes in apple, particularly in the context of cell dedifferentiation remains scarce. In this study, we confirmed the positive effect on *MdMAN7*-mediated cell wall degradation in cell dedifferentiation and SE in apple through genetic overexpression and knockout approaches. Additionally, although no overt morphological abnormalities were observed in the tissue-cultured transgenic plantlets of miR3699 or *MdMAN7*, based on their roles in cell wall remodeling, we speculate that progeny of the transgenic lines may differ from wild-types in aspects such as stress tolerance and floral organ development. For instance, alterations in cell wall architecture may regulate the release of defense-eliciting oligosaccharides, thereby enhancing resistance against pathogens [44]. Changes in cell wall porosity and water-holding capacity could influence drought tolerance [45]. Pectin modification has been shown to affect flowering time in plants [46].

**Figure 9:**
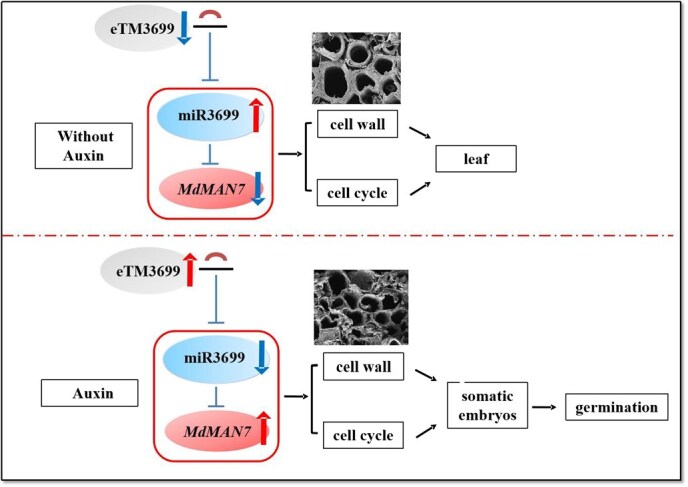
Model illustrating the role of miR3699, MdMAN7, and eTM3699 during the formation of embryogenic cells and SE in apple.

The ceRNA networks have been proved to regulate SE in plants. For examples, SE is regulated by the lncRNA125175–miR393h–TIR2 network through auxin signaling in garlic [47] and by the eTM171–miR171–SCL6 in *Lilium pumilum* DC. Fisch [19], respectively. The eTM–miR160–*ARF10* pathway regulating SE in *Dimocarpus longan* Lour [48]. In this study, miR3699 was predicted to target *MdMAN7*, and lncRNA MSTRG.1226.2 (eTM3699) was predicted to function as an eTM for miR3699 through whole-transcriptome sequencing. The function of miR3699 in SE in plants remains unclear. This study firstly validated the accuracy of the eTM3699–miR3699–*MdMAN7* module using dual-luciferase and GUS assays. Subsequently, the function of miR3699 in SE in apple was proved through stable overexpression and CRISPR-Cas9 mediated transgenic assays, which the miR3699 can negatively regulate embryogenic cell formation and SE by targeting *MdMAN7*. Under the induction of exogenous auxin, eTM3699 competitively binds to miR3699, thereby inhibiting the cleavage of the targets *MdMAN7* by miR3699. The elevated expression level of *MdMAN7* enhanced the activity of β-mannanase, leading to cell wall degradation, which alleviates the constraints on somatic cell dedifferentiation and consequently promotes SE in apple (Fig. 9). This study advances our understanding of somatic cell reprogramming in apple and provides insights for germplasm enhancement and molecular breeding of this economically important fruit crop.

## Materials and methods

### Plant material and treatment

The aseptic seedlings of *M. domestica* ‘Gala’ were grown in a plant tissue culture laboratory under 16/8 h light/dark conditions at 24 ± 2°C. Leaves of the plants were placed in EIM (MS semisolid medium with 0.5 mg·l^−1^ 2,4-D and 3.0 mg·l^−1^ NAA, pH 5.8 ± 0.2) with a diameter of 1 × 2 cm (rectangular cutting) in the dark for 8 days to induce embryogenic cell formation. The Day 8 leaves were subsequently placed in SEM (MS semisolid medium with 1.0 mg·l^−1^ 6-BA, pH 5.8 ± 0.2) under darkness for 30 days to induce mature somatic embryos. The mature somatic embryos were subsequently cultured under 16/8 h light/dark conditions for 30 days until germination in SEM. Tissue and cytological observations were performed on leaves cultured for 0–8 days in EIM. Six-day embryogenic leaves grown in EIM were used as receptors for genetic transformation.

### Physiological-biochemical assay

D-Mannose (CAS: G0583W) and hemicellulose (CAS: G0716W) content in leaves were analyzed using commercial assay kits (Grace, Suzhou). β-Mannanase (CAS: G0716W) and hemicellulose (CAS: G0709W) activity were measured following the manufacturer’s protocols, respectively.

### Electron microscope observation

The Day 0 and Day 8 leaves which cultured in EIM of *M. domestica* ‘Gala’ were fixed in 2.5% glutaraldehyde at 4°C overnight. After rinsing with 0.1 M PBS buffer (pH = 7.0) three times, the samples were dehydrated with gradient concentration of ethanol, and then sufficiently dried. The Cressington 108Auto coater was used to coat samples and the scanning electron microscope (GeminiSEM 360) and transmission electron microscope (HITACHI HT7700) were used to observe cells. The thickness of cell walls and the number of cells were quantified using ImageJ software.

### LncRNA and mRNA data analysis

Following extraction with TRIzol Reagent (Invitrogen), RNA quality was confirmed spectrophotometrically (Infinite® 200 PRO; Tecan, Switzerland). High-quality RNA was reverse-transcribed into cDNA (M-MLV RTase Kit, TaKaRa, D6130), followed by construction of six cDNA libraries (Illumina TruSeq Stranded mRNA Kit, RS-122-2101). Novel transcripts were analyzed for protein-coding potential using CNCI (v2) and CPC (v0.9-r2) with default parameters [49]. The intersection of nonprotein-coding predictions derived from dual analytical approaches was designated as lncRNAs. Transcript quantification was performed through reference-guided analysis using STRINGTIE (v1.3.4d). The *M. domestica* HFTH1 Genome v1.01 served as the reference for mRNA transcriptome alignment.

### miRNA data analysis

Following total RNA extraction (TRIzol, Invitrogen), small RNAs (18–30 nt) were PAGE-fractionated and ligated to 3′ and 5′ adapters for library construction. After adapter ligation, RNAs (36–44 nt) were reverse-transcribed and PCR-amplified. cDNA libraries (140–160 bp) were size-purified and sequenced on an Illumina NovaSeq X Plus platform (GeneDenovo, Guangzhou).

### Differential expression analysis across miRNA, mRNA, and lncRNA types

Differential expression analysis was performed with: For miRNA, edgeR (|log₂FC| ≥ 2, *P* < 0.05). For lncRNAs and mRNAs, DESeq2 (FDR < 0.05, |log₂FC| > 1). Target genes were predicted using patmatch_v1.2. Significant DE miRNAs, mRNAs, and lncRNAs were functionally annotated via GO and KEGG enrichment analysis.

### Constructing and analyzing the ceRNA network

An lncRNA–miRNA–mRNA ceRNA network was built according to the Salmena *et al.* [24] hypothesis, which proposes that lncRNAs compete for miRNA binding as eTMs. The _PS_RNAT_ARGET_ algorithm [50] was employed to forecast potential interactions between lncRNA–miRNA and miRNA–mRNA. The Spearman correlation coefficient (SCC) was calculated to assess expression correlations for lncRNA–miRNA and miRNA–mRNA pairs. Pearson correlation coefficient (PCC > 0.7) for lncRNA–mRNA co-expression. Co-expressed lncRNA–mRNA pairs (PCC > 0.7) that were both negatively correlated with a shared miRNA were retained. The final network was plotted in Cytoscape [51].

### RT-qPCR

RT-qPCR was performed using a QuantStudio 5 system (Applied Biosystems). Total RNA was extracted following a standard protocol for transcriptome analysis. For mRNA and lncRNA, the first-strand cDNA was synthesized using a M-MLV RTase cDNA Synthesis Kit (Catalog No. D6130; TaKaRa). The reaction mixture volume was 20 μl, including total RNA 1 μg, 5× gDNA Buffer 2 μl, 10× RT Buffer 2 μl, RT Enzyme 1 μl, RT Primer Mix 2 μl, and make up to 20 μl with RNase-free water. The RT-PCR procedure was as follows: 42°C for 15 min and 95°C for 3 min. The cDNA was diluted with RNase-free water to approximately 200 ng/μl for RT-qPCR. The RT-qPCR for mRNA was performed as the description of SYBR Premix Ex TaqTM II (TaKaRa, Dalian, China). Each reaction mixture volume was 20 μl, including 1 μl of cDNA, 0.5 μl of forward primer, 0.5 μl of reverse primer, 10 μl of Ultra SYBR mixture, and 8 μl of RNase-free water. The RT-qPCR procedure was as follows: after starting at 95°C for 30 s, the fusion curve was analyzed after 45 cycles of 95°C for 30 s, 58°C for 15 s, and 72°C for 30 s. The RT-qPCR for lncRNA was performed as the description of lnRcute lncRNA qPCR Kit (Tiangen, Beijing, China). Each reaction mixture volume was 20 μl, including 1 μl of cDNA, 0.5 μl of forward primer, 0.5 μl of reverse primer, 10 μl of lnR lncRNA PreMix, and 8 μl of RNase-free water. The RT-qPCR procedure was as follows: after starting at 95°C for 3 min, the fusion curve was analyzed after 45 cycles of 95°C for 5 s, 58°C for 10 s, and 72°C for 15 s. The *18S* rRNA (DQ341382) was selected as the internal reference gene [23].

For mature miRNA analysis, the miRcute Plus miRNA cDNA Kit (Tiangen) was used to synthesize the first-strand cDNA. The reaction mixture volume was 20 μl, including total RNA 1 μg, 2× miRNA RT Reaction Buffer 10 μl, miRNA RT Enzyme 2 μl and make up to 20 μl with RNase-free water. The RT-sPCR procedure was as follows: 42°C for 60 min and 95°C for 3 min. The RT-qPCR for miRNA was performed as the description of miRcute Plus miRNA qPCR Kit (Tiangen). Each reaction mixture volume was 20 μl, including 1 μl of cDNA, 0.5 μl of forward primer, 0.5 μl of reverse primer, 10 μl of miRNA PreMix and 8 μl of RNase-free water. The RT-qPCR procedure was as follows: after starting at 95°C for 15 min, the fusion curve was analyzed after 45 cycles of 95°C for 20 s and 60°C for 35 s. The *5.8S* rRNA (DQ341382) was selected as the internal reference gene [23]. Gene expression was quantified via the 2^−ΔΔCt^ method with three biological and technical replicates.

### Plasmid construction and transformation

The pre-miR3699 sequence and the full-length coding regions of *MdMAN7* were amplified and ligated into the pCambia2300 vector, yielding overexpression vectors. Two mutation targets for *MdMAN7* were designed via an online tool (https://cctop.cos.uni-heidelberg.de:8043/index.html). The method of plasmid construction was described previously [52]. The procedures used for overexpression of miR3699 and *MdMAN7* and CRISPR/Cas9-mediated gene editing of *MdMAN7* by agroinfiltration in *M. domestica* ‘Gala’ were described previously [52]. The fragment of *MdMAN7* were cloned and sequenced by Tsingke (Shanxi, Xi’an), and DNAMAN software was used to analyze the mutation types. Statistics on the induction rate and germination coefficient of the transformed lines were analyzed. The SE induction rate (%) = number of induced explants/total explants × 100%. The germination coefficient = the number of regenerated plants on each explant leaf. The media recipes reference Table S1.

### Dual-luciferase reporter assay

The dual-luciferase reporter assays were used to confirm the interaction between miR3699, *MdMAN7*, and eTM3699. *MdMAN7* and its synonymous mutation sequence (*MdMAN7-M*) were inserted into the pGreenII 0800-LUC reporter vector. The pre-miR3699 sequence and the fragments of eTM3699 were inserted into the pGreenII 62-SK vector. Transformation and infiltration methods were described previously [52]. The plant molecular imaging system (LB985 NightSHADE) was used to observe the fluorescents.

### GUS assay

The GUS histochemical staining assays were used to confirm the relationship between miR3699, *MdMAN7*, and eTM3699. The full-length coding region of *MdMAN7* was inserted into the pBI121 vector. The pre-miR3699 sequence and the fragments of eTM3699 were inserted into the pBI121 vector to replace the GUS sequence, respectively [53]. Transformation and infiltration methods were performed as described by Jefferson *et al*. [54]. The GUS content of stained tissue was measured using a GUS-ELISA Kit (MLBio, Shanghai, China).

### Data analysis

Data were analyzed with GraphPad Prism (v10.2.3) and Microsoft Excel 2019. Experiments included three biological and technical replicates, with results presented as mean ± SE. Differences between groups were assessed for significance by Student’s *t*-test (^*^P < 0.05, ^**^P < 0.01). The primer sequences reference Table S5.

## Supplementary Material

Web_Material_uhaf315

## Data Availability

The transcriptome data involved in this study have been uploaded to NCBI under number PRJNA1296799.
