# Supplementary material for: The eTM–miR3699–MAN7 mediated cell wall degradation in regulating embryogenic cell formation during the early stage of somatic embryogenesis in apple
Source: Hortic Res. 2025 Nov 14;13(2):uhaf315. doi: 10.1093/hr/uhaf315 (PMC12946679; doi:10.1093/hr/uhaf315)
Supplement: Web_Material_uhaf315 [file web_material_uhaf315.zip › Supplemental Figures.docx]

**The eTM-miR3699-MAN7 mediated cell wall degradation in regulating embryogenic cell formation during the early stage of somatic embryogenesis in apple**

Yue Yang^1†^, Yu Wang^1†^, Mingkun Chen^1†^, Xilin Zhou^2^, Jun Wei^1^, Jiayao Tang^1^, Houhua Li^1*^

^1^*College of Landscape Architecture and Art, Northwest A&F University, Yangling 712100, Shaanxi, China*

^2^*College of Horticulture and Forestry, Tarim University, Alar, Xinjiang 843300*

Yue Yang: 2023130012@nwafu.edu.cn

Yu Wang: 2285588754@qq.com

Mingkun Chen: [fafucmk@163.com](mailto:fafucmk@163.com)

Xilin Zhou: [1504010045@qq.com](mailto:1504010045@qq.com)

Jun Wei: [3353734603@qq.com](mailto:3353734603@qq.com)

Jiayao Tang: [3078374825@qq.com](mailto:3078374825@qq.com)

Corresponding author：Houhua Li

^*^ Correspondence: [lihouhua@nwafu.edu.cn](mailto:lihouhua@nwafu.edu.cn)

Tel: +86 15114800050

**Supporting Information**

**
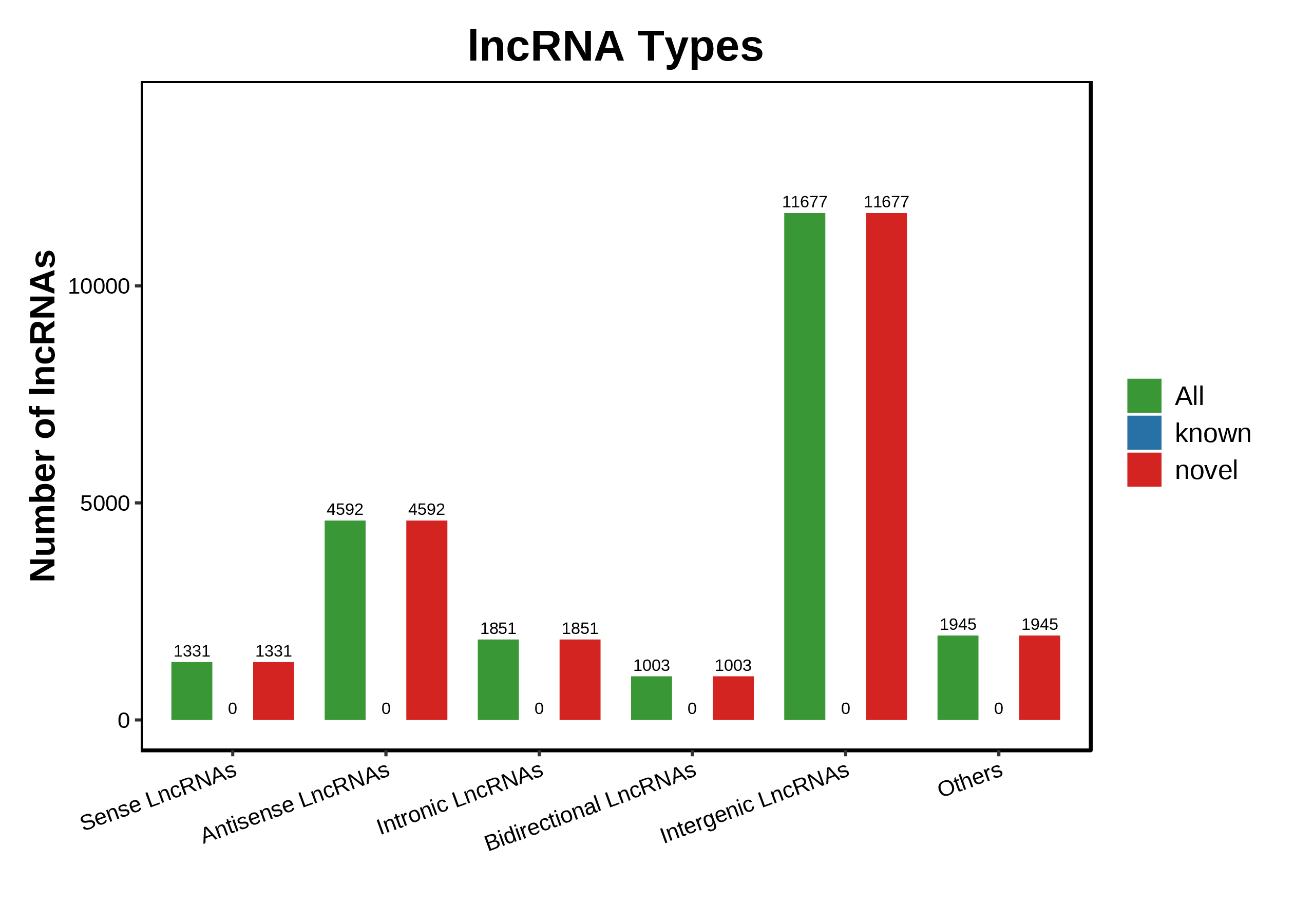
**

**Figure S1.** Classification of lncRNAs.

**
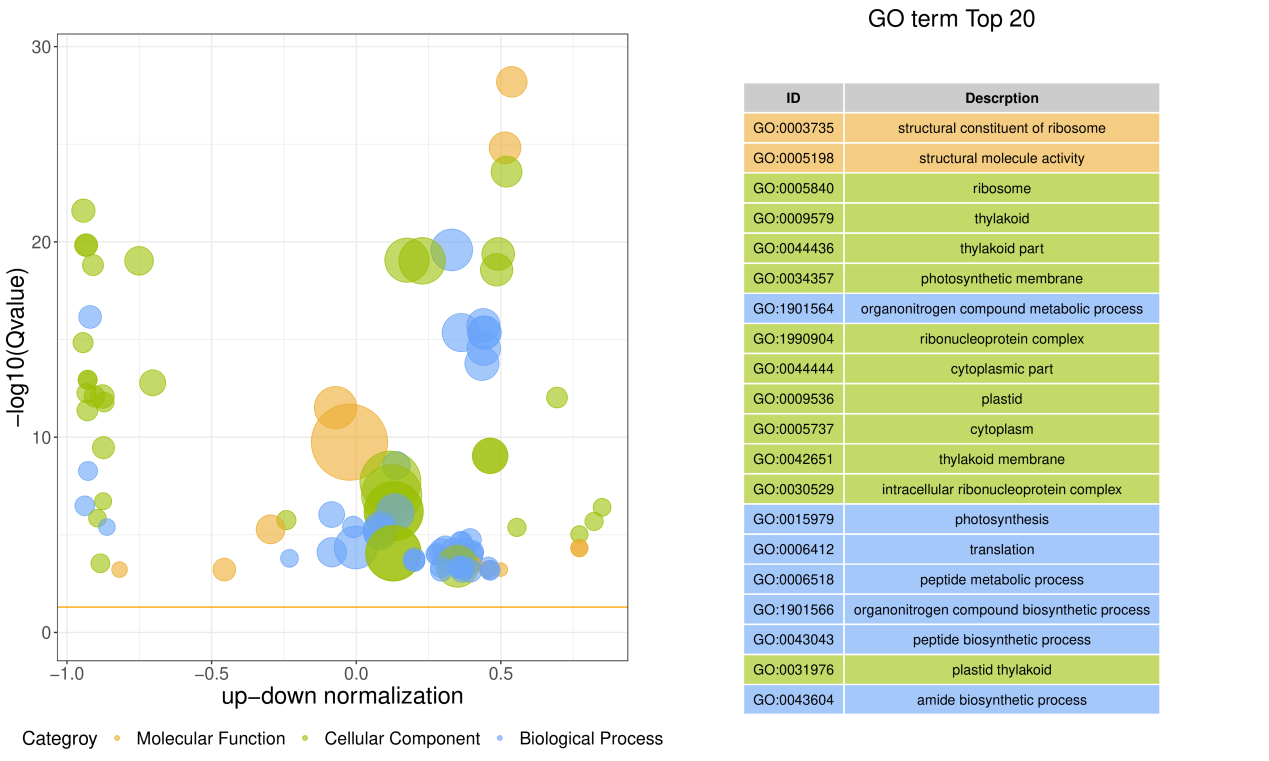
**

**Figure S2.** Gene Ontology (GO) enrichment analysis of DEmRNAs.

**
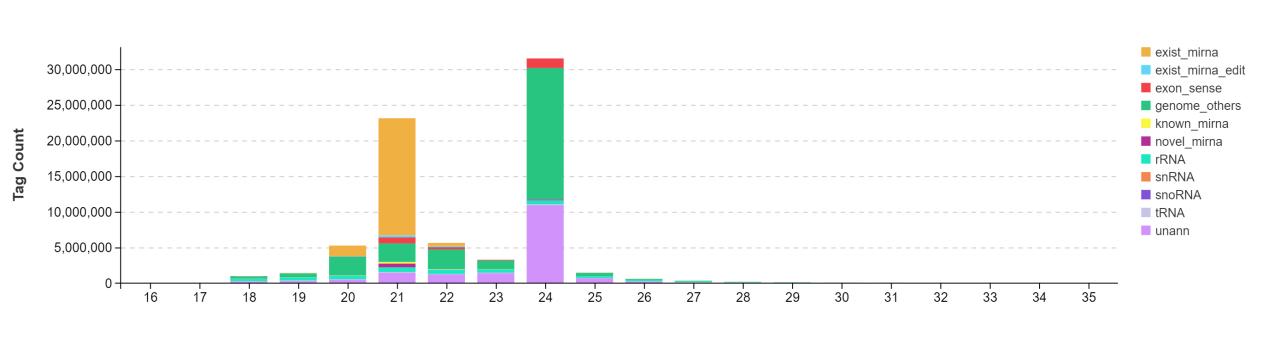
**

**Figure S3.** The length distribution analysis of miRNAs in *Malus* *domestica* ‘Gala’ leaves.


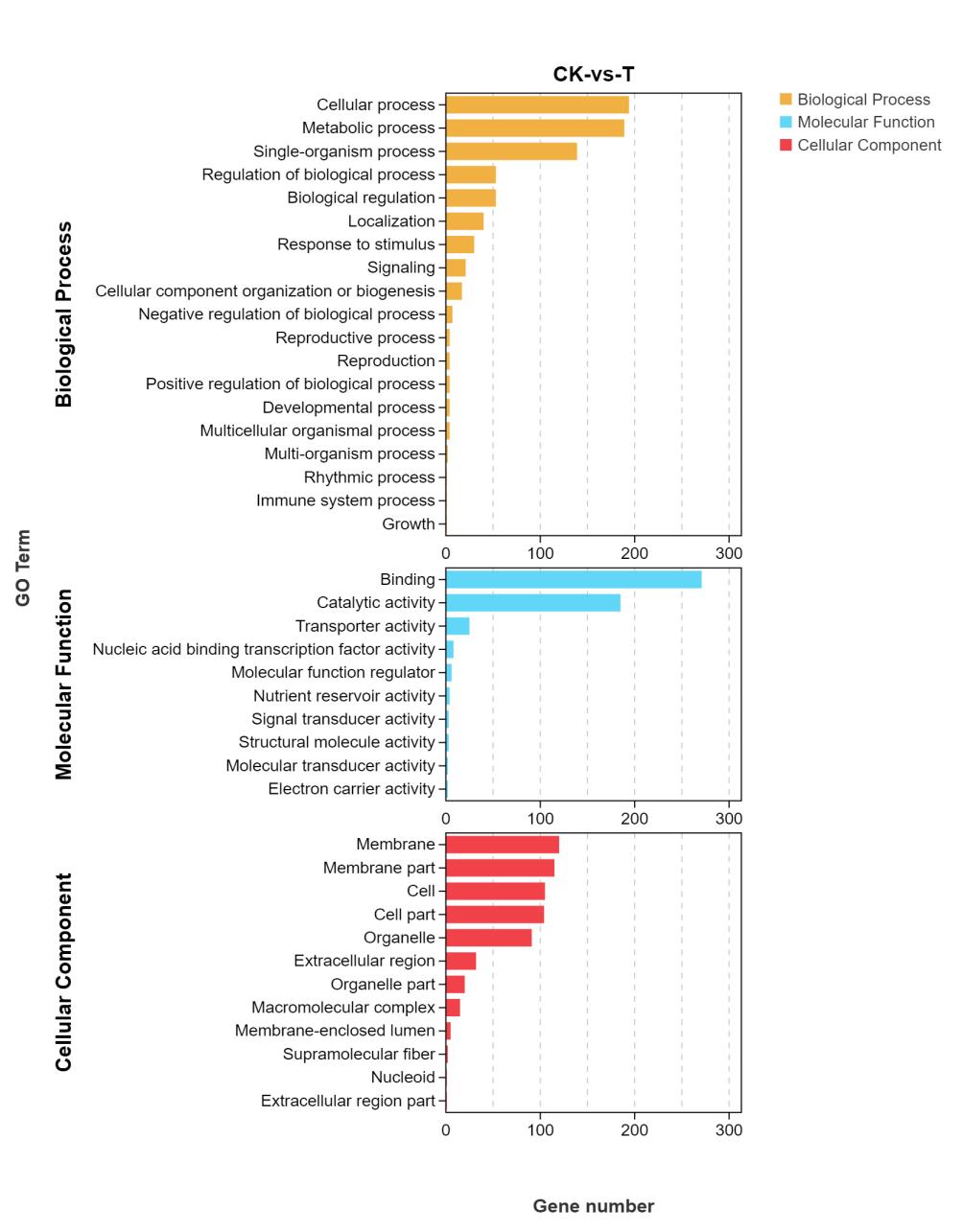


**Figure S4.** GO enrichment of DEmRNAs targeted by DEmiRNAs.


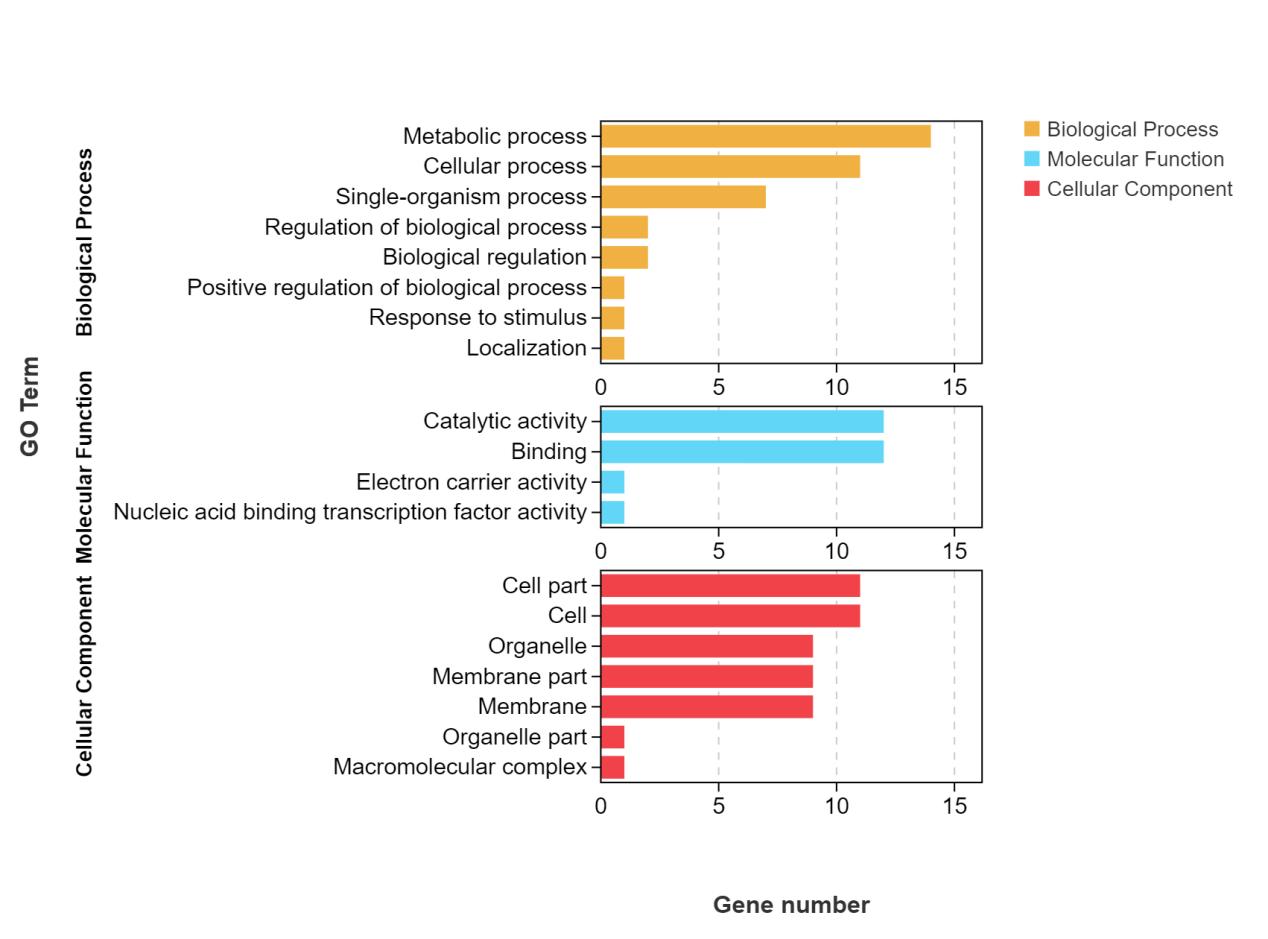


**Figure S5.** GO enrichment of ceRNAs.

**
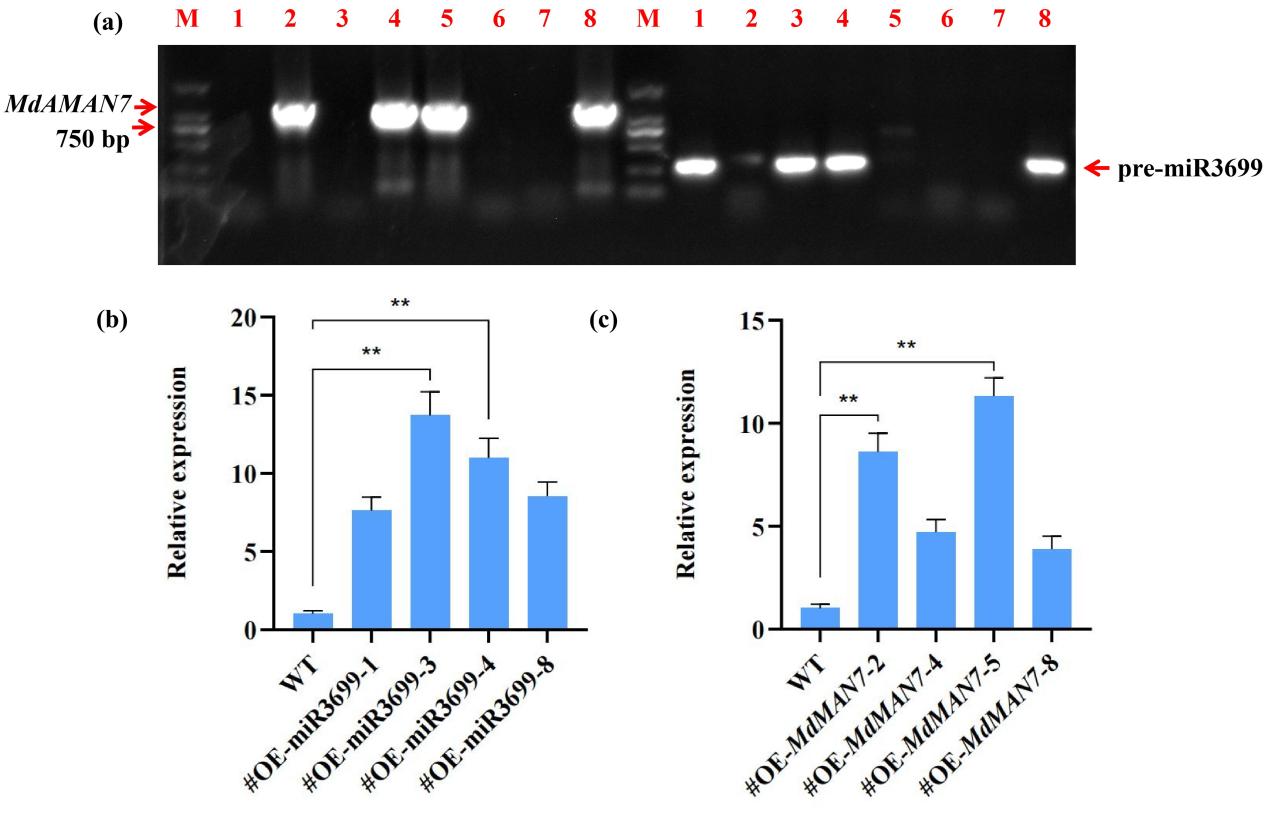
**

**Figure S6.** Identification of miR3699*-* and *MdMAN7*-overexpressing lines. (a) PCR detection of miR3699*-* and *MdMAN7*-overexpressing resistant plants. (b) Expression levels of miR3699 in the miR3699-overexpressing lines. (c) Expression levels of *MdMAN7* in *MdMAN7*-overexpressing lines. The values represent the means ± SEs. Asterisks indicate statistically significant differences, as determined by Student’s *t*-test (* *P*<0.05, ** *P*<0.01).

**
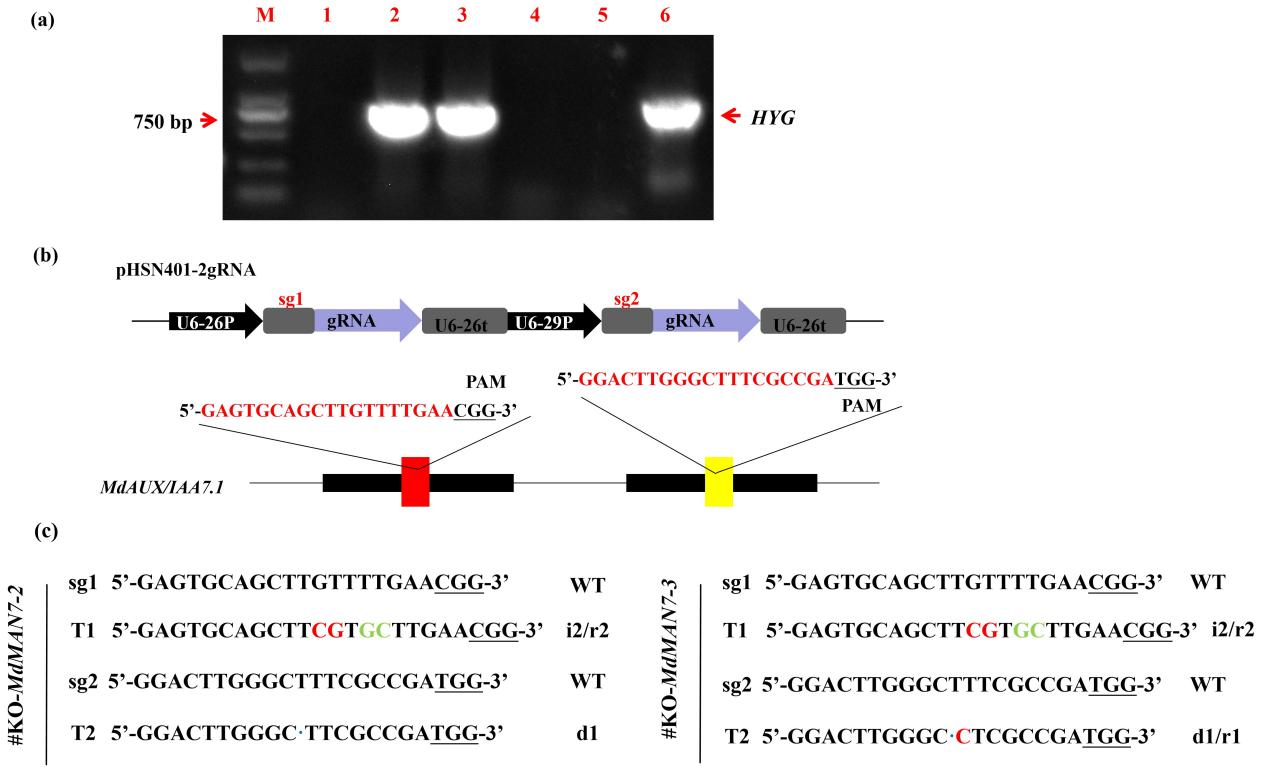
**

**Figure S7.** *MdMAN7* gene editing in apple. (a) PCR detection of *HYG* in resistant plants. (b) Typical CRISPR/Cas9-mediated *MdMAN7* editing vector (pHSN401-2gRNA) construct. (c) *MdMAN7* mutation sites analysis in #KO-*MdMAN7*-2/3. Green, nucleotide insertion. Red, nucleotide replacement. Blue, nucleotide deletion.
